# General practitioners’ involvement in inpatient medical rehabilitation in Germany: a scoping review

**DOI:** 10.1186/s12875-025-03007-5

**Published:** 2025-09-18

**Authors:** Malte Klemmt, Katharina van Baal, Maria Bonin, Stephanie Stiel

**Affiliations:** Institute for General Practice and Palliative Care, Hannover Medical School, Carl-Neuberg-Straße 1, 30625 Hanover, Germany

**Keywords:** General practitioner, Medical rehabilitation, Inpatient rehabilitation, Germany, Scoping review

## Abstract

**Background:**

The German rehabilitation system has some peculiar features, such as the predominance of inpatient medical rehabilitation. Most patients receive inpatient rehabilitation due to chronic illnesses like chronic backpain or psychosomatic diseases. General practitioners play a special role in the German healthcare system as they are often the first point of contact for health issues. The study aims to provide an overview of the involvement of general practitioners in inpatient medical rehabilitation in Germany as well as identifying barriers and facilitators. By doing this, a basis for optimizing general practitioners' involvement can be generated.

**Methods:**

A scoping review was conducted to ascertain the current state of scientific knowledge, employing the methodological approach of the Joanna Briggs Institute. The search was carried out between February and March 2024. Eight databases were screened: PubMed, Web of Science Core Collection, PubPsych, EBSCOhost, BeLit, LIVIVO, ProQuest, and German National Library. Publications in German and English in the publication period from 1980 to February 2024 were sought. A thematic analysis was conducted to evaluate the included publications. The research process and generation of findings were recorded utilizing the PRISMA-ScR checklist.

**Results:**

The search resulted in a total of 2231 records, of which 102 were screened on a full text level. Fifty-four publications were included in the review. Following the thematic analysis, fourteen themes were identified and assigned to four topic areas. In the area of access, general practitioners take on a gatekeeper function (addressed by 10 publications) among other things. In follow-up care, there is involvement in prescription (5 publications), like referral to outpatient medical specialists. Barriers to involvement relate, for example, to a lack of knowledge on the part of general practitioners (10 publications) and facilitators include education and training (10 publications).

**Conclusions:**

The results confirm the important role of general practitioners in providing access to inpatient medical rehabilitation in Germany and follow-up care. However, various barriers for general practitioners' involvement were revealed. In order to maintain and optimize the involvement, further scientific research and efforts based on this in practice are necessary, involving relevant stakeholders.

## Background

Medical rehabilitation is an important part of healthcare at various stages of life if there is a risk of or already an existing health-related impairment to social and occupational participation [1]. Due to the high prevalence of chronic diseases, residual illnesses, and the changing demographic structure, medical rehabilitation is of great importance for reintegrating patients into work and society [2]. In Germany, medical rehabilitation mainly takes place on an inpatient basis, where patients typically spend a period of at least 3 weeks. In some cases, such as mental illnesses like depression, this period can be extended up to seven weeks [3]. The objective of inpatient medical rehabilitation (IMR) is to create an environment that helps the patient to fully focus on recovery and rehabilitation. Due to the availability of specialized clinics, the rehabilitation stay is often outside of the patients' hometown [3]. Also, rehabilitation clinics in Germany are highly specialized and focus on specific medical indications. Medical indications in which IMR most frequently take place are (in order of frequency): orthopedics, psychosomatics/psychotherapy, oncology, cardiology, neurology, addiction disorders, and pulmonology/pneumology [4]. In 2023, there were over 1.000 rehabilitation clinics with more than 161.000 beds in Germany. The size of the clinics varies from 30–40 beds up to clinics with over 500 beds. The average patient occupancy rate in 2023 was 81.5% [5]. In the rehabilitation clinics, interdisciplinary teams encompass various professionals (e.g. rehabilitation physicians, physiotherapists, occupational therapists, nutritionists, psychologists) with a strong medical dominance [3]. In Germany, IMR is financed either by the German pension insurance, the statutory health insurance or an accident insurance [6]. For people of working age (usually 18–67 years old), IMR is typically financed by the German pension insurance. This is the case for up to two thirds of all rehabilitation procedures [7]. Almost 1.5 million rehabilitation applications were submitted to the German pension insurance in 2022 [4]. Patients have to submit an application for IMR by themselves [6]. Almost 70% of patients come to IMR from their home environment due to a chronic illness such as chronic back pain or psychosomatic diseases. Direct follow-up treatment following a hospital stay (e.g. after a heart attack or stroke) occurs in slightly less than 30% of the cases [4]. Following the inpatient rehabilitation stay, patients can take advantage of outpatient follow-up care services to pursue their long-term rehabilitation goals [8].

General practice is characterized by a long-lasting doctor-patient relationship with frequent contacts. As a result, general practitioners (GPs) are usually aware of their patients' medical biographies, mental vulnerability, their social network, and occupational situation [9]. Moreover, GPs commonly care for all age groups and social classes and treat a wide range of illnesses, which means that they can reach various target groups [10]. In addition to providing medical care to their patients, GPs in Germany operate as gatekeepers to other services in the healthcare system. Therefore, GPs are responsible for identifying the care needs of their patients and referring them to inpatient or outpatient care as required [11]. Given this role, GPs are ideally the first point of contact as well as advisors and coordinators regarding the IMR [9]. These forms of GPs' participation (e.g. as support in the application process) are demanded at a political level [12], endorsed by the German pension insurance [13], and desired by patients [14].

However, there are implications that the involvement of GPs in the IMR process needs to be optimized [15]. Known optimization potentials concern, for example, information deficits among GPs [16], limited awareness of the importance of rehabilitation in general [17], or interface problems between GPs and rehabilitation clinics [18]. Insufficient involvement of GPs in IMR could jeopardize the principle of needs-based access to rehabilitation [2] and the long-term maintenance of positive treatment effects [19]. In Germany, no specific GP guidelines exist regarding rehabilitation [6].

To date, there has been no overview of the forms and characteristics of GPs' involvement in the German rehabilitation setting. It is unknown how relevant stakeholders perceive GPs' involvement and what problems may exist. This study aims to fill this gap and answer the following research questions:

- To what extent and how are GPs involved in IMR in Germany?
- What facilitators and barriers exist concerning GPs' involvement?

The generated overview on GPs' involvement in IMR will provide gathered evidence that can serve as a basis for improving the quality of IMR in Germany by involving GPs more effectively into the rehabilitation process. Different forms of involvement and areas in which GPs are (not) involved may be identified and reported barriers can be addressed, allowing for a better coordination of care, higher patient satisfaction, a more needs-oriented care and ultimately better health outcomes for patients. The results of this review might also help to improve the education and training of GPs to better prepare them for their role in IMR. Moreover, international health care systems might benefit from the results of this review. Through comparison, international researchers and political decision-makers can learn from German experiences and challenges with GPs' involvement in IMR. This review allows a comparison of challenges and adaptation of possible solutions for countries or systems in which GPs have a similar position. International researchers and professionals from countries in which GPs are not or less involved in IMR can gain insights into IMR in Germany and facilitators and barriers concerning GPs' involvement.

## Methods

Due to the exploratory character of the research questions, a scoping review methodology was chosen to review existing literature on GPs' involvement in IMR in Germany. A scoping review encompasses the mapping of pertinent scientific knowledge and allows normative-theoretical literature to be collected alongside empirical studies [20]. We conducted a scoping review following the guidelines proposed by Peters et al. from the Joanna Briggs Institute (JBI) [20]. The guidelines include the following nine steps:

1. Defining and aligning the objective and question
2. Developing and aligning the inclusion and exclusion criteria with the objective and question
3. Describing the planned approach to evidence searching, selection, data extraction, and presentation of the evidence
4. Searching for the evidence
5. Selecting the evidence
6. Extracting the evidence
7. Analyzing the evidence
8. Presenting the results
9. Summarizing the evidence in relation to the purpose of the review, drawing conclusions, and noting any implications of the findings

A review protocol was prepared in advance, which can be requested from the corresponding author. The PRISMA-ScR checklist [21] was adopted to document the review process and report the findings (see additional file 1).

### Inclusion and exclusion criteria

The search was limited to publications in German and English with a publication date from 1980 until February 2024. The decision was based on a preliminary search in which the earliest publication was published in 1980. Both empirical and theoretical publications were considered, including journal articles, monographs, dissertations, and book chapters. Reviews, abstracts, and conference contributions were excluded. Inclusion criteria in terms of the publications' content followed the JBI differentiation of population (GPs), concept (all forms of participation in IMR), and context (IMR in Germany across all indications for adults, excluding rehabilitation for children and adolescents, because typically GPs are only responsible for patients from adulthood onwards). Only publications concerned with GPs' involvement in IMR in the German context were considered.

### Search

One author (MK) conducted the search based on the three-stage search strategy of the JBI method [20]:

1. keywords and index terms for database search
2. iterative searches in databases through refining and adapting
3. screening of reference lists of the included publications after removing duplicates

Eight databases (PubMed, Web of Science Core Collection, PubPsych, EBSCOhost, BeLit, LIVIVO, ProQuest, and German National Library) were searched for relevant publications in February and March 2024. A search strategy for each database was developed beforehand. Table 1 contains the specific search terms in German and English and an exemplary search string for the database PubMed.

**Table 1:** Search terms and exemplary search string

	***German***	***English***
***Population/Setting***	Hausarzt/Hausärztin Hausarztpraxis Allgemeinmedizin Allgemeinmediziner:in	general practitioners general practice family practice general medicine family medicine primary health care primary care family doctor family physician primary care physician
***Context***	Rehabilitation Medizinische Rehabilitation Stationäre Rehabilitation Rehabilitationsmedizin	rehabilitation medical rehabilitation inpatient rehabilitation rehabilitative medicine
***Concept***	Beteiligung Involviertheit Einbeziehung Einbindung Engagement Förderfaktoren förderliche Faktoren Barrieren Hinderliche Faktoren Schwierigkeiten Herausforderungen Probleme	involvement participation integration commitment engagement promotion factors Support factors barriers hindering factors difficulties challenges problems

Exemplary search string (PubMed):

("general practitioners"[MeSH Terms] OR "general practice"[MeSH Terms] OR “family practice”[MeSH Terms] OR "general medicine"[Title/Abstract] OR “family medicine” [Title/Abstract] OR "primary health care"[MeSH Terms] OR “primary care”[Title/Abstract] OR "family doctor"[Title/Abstract] OR “physicians, family"[MeSH Terms] OR "physicians, primary care"[MeSH Terms]) AND ("rehabilitation"[MeSH Terms] OR "inpatient rehabilitation"[Title/Abstract] OR "medical rehabilitation"[Title/Abstract] OR “rehabilitative medicine”[Title/Abstract]) AND ("involvement"[Title/Abstract] OR "participation"[Title/Abstract] OR "integration"[Title/Abstract] OR "commitment"[Title/Abstract] OR "engagement"[Title/Abstract])

Additionally, ten publisher catalogues (Elsevier/ScienceDirect, Springer Link, Oxford Academics, Thieme online, De Gruyter, Hogrefe, SAGE, Taylor & Francis, Cambridge University Press-Core, and Beck-online) were screened for publications eligible for inclusion.

### Selection

The publication selection comprised several phases. Identified publications were first screened on title and abstract level by the first author (MK) regarding relevance and fulfillment of the inclusion criteria. The remaining publications were screened for duplications. After that, reference lists of the remaining publications were screened and additional publications were included if they met the inclusion criteria. Lastly, two authors (MK, KvB) independently performed the full text screening of each publication for relevance and compliance with the inclusion criteria. In case of discrepancies between the two reviewers (interrater reliability: 80.4%), a third author (MB) was consulted and decided on inclusion.

### Data extraction and analysis

The data for the analysis was gained from the results and discussion sections of the included publications. The (interpretative) results (e.g. statistical correlations) and, if available, original data (e.g. interview statements) were incorporated from both quantitative and qualitative study designs (or mixed methods approaches) and theoretical studies (arguments). A thematic analysis according to Braun & Clarke [22] was performed to evaluate the data, comprising the following phases:

1. familiarizing with the data
2. generating initial codes
3. searching for themes
4. reviewing themes
5. defining themes
6. reporting

Components of the included publications (e.g. a survey result or interview statements) that are relevant to the research question of the review were coded and assigned to themes. For example, a survey result in which GPs stated that they had too little knowledge about rehabilitation to be able to recognize eligible patients was assigned to the topic of barriers respectively lack of knowledge. Phases 2–5 were conducted using the MaxQDA software (version 18). The initial analysis was carried out by one author (MK). All authors were involved in the assessment, validation, and critical reflection of the analysis results as well as in the interpretation of the findings. The characteristics of the included publications were reported with frequencies and percentages. The same applies to quantitative data relating to the identified themes.

## Results

### Characteristics of the included publications

**Fig. 1:**
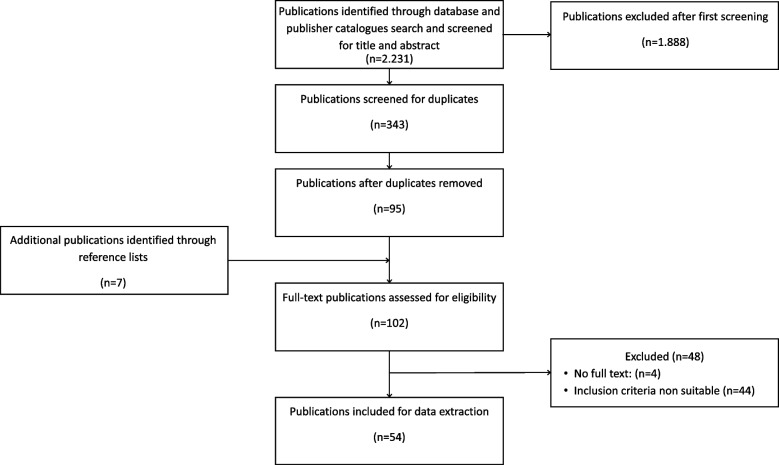
Flowchart

Fifty-four publications met the inclusion criteria. Figure 1 shows the study inclusion process.

Forty-five (83%) of the included publications are empirical studies, nine (17%) are theoretical publications. The study designs of the empirical studies are mostly (*n* = 32, 71%) quantitative (e.g. cross-sectional survey, randomized controlled trial, document analysis, observational study), 9 (20%) publications have a qualitative study design (e.g. interview study, focus group) and four (9%) publications have a mixed methods design. The theoretical publications deal with the perspectives of GPs, outpatient specialists, or a legal or systemic perspective. Thirty-four (63%) publications are cross-indication oriented, specifically addressing orthopedics (*n* = 8, 15%), psychosomatics (*n* = 4, 7%), neurology (*n* = 4, 7%), cardiology (*n* = 3, 6%), and addictive disorders (*n* = 1, 2%). The range of the publication years is 1989 to 2023. Table 2 provides an overview of the publications included.

**Table 2:** Overview of the included publications

Authors	Year	Title	Indication	Aim	Study design	Main results regarding the topic of the review
Ahnert et al	2018	[Application for inpatient psychosomatic rehabilitation: frequency, quality, and approval rates]	Psychosomatics	Examination of formalities and approval criteria for rehabilitation applications in psychosomatic patients	Quantitative: prospective document analysis (1.366 applications for rehabilitation)	• 44% of applications for psychosomatic IMR are accompanied by a diagnostic report from the GP • Applications accompanied by a diagnostic report from a specialist are approved more frequently than applications accompanied by a diagnostic report from a GP
Barzel et al	2008	[Outpatient management of stroke patients from the viewpoint of general practitioners in Hamburg—an exploratory study]	Neurology	Evaluation of the quality of care of stroke patients after the acute treatment	Quantitative: cross-sectional survey (443 GPs)	• GPs mainly prescribe medication and physiotherapy after neurological IMR • There are different attitudes among GPs with regard to the success of neurological IMR and follow-up care
Best et al	2015	[Practically relevant cooperation and networking in rehabilitation]	Orthopedics	Provision of an overview of socio-medical and socio-legal principles of rehabilitation	Theoretical (perspective: social medicine & social law)	• GPs mainly prescribe training and physiotherapy after orthopedic IMR, they are also responsible for monitoring the procedures and effects
Brandt	1989	[Medical rehabilitation from the perspective of the general practitioner]	Across indications	Examination of the role of office physicians in the rehabilitation system	Quantitative: cross-sectional survey (775 GPs and 127 orthopedists in private practice)	• GPs have a rather positive attitude towards IMR in general • Identified lack of knowledge with regard to IMR • Deficits in cooperation between the stakeholders involved
Burchadi et al	2023	Effectiveness of a screening tool to assess prevention and rehabilitation needs of 45 to 59 years old in primary care—study protocol of a pragmatic randomized controlled trial (PReHa45)	Across indications	Evaluation of an intervention to support GPs in identifying rehabilitation needs and submitting applications	Study protocol	• Importance of GPs in accessing IMR
Deck et al	2000	[Interface problems in medical rehabilitation: development of a brief questionnaire to assess GPs needs for information and communication]	Across indications	Development of a questionnaire to assess GPs needs for information and communication in the rehabilitation context	Quantitative: cross-sectional survey (98 GPs and specialists in private practice)	• 61% of GPs rate the outcome of IMR as good • Many GPs experience difficulties in working with the rehabilitation clinic • The more positive GPs are about IMR, the more likely they are to recommend IMR to their patients
Deck et al	2009	[Identification of potential need for medical rehabilitation by general practitioners: idea and reality]	Across indications	Identification of potential need for rehabilitation by GPs through a checklist	Quantitative: cross-sectional survey (60 patients) and observations via checklist (9 GPs)	• When assessing their patients' need for MIR, GPs tend to focus on disease-related characteristics • GPs show negative attitude towards IMR and lack of knowledge
Deck et al	2009	[Improvement of rehabilitation aftercare through long term follow along of the patients – results of a pilot study]	Orthopedics	Development and evaluation of a new strategy and organization of follow-up care in patients with chronic low back pain	Mixed methods: cross-sectional survey (77 patients) and expert discussions (19 members of rehabilitation staff)	• Information materials on IMR for GPs are evaluated positively
Deck et al	2018	[Digital information on rehabilitation and retirement for physicians—a practical test]	Across indications	Evaluation of an information website for GPs	Mixed methods: pre-post survey and think-aloud-technique (79 postgraduate GP trainees)	• There is generally a great need for information among GPs with regard to IMR
Deventer	2007	[Initiating, applying for or prescribing a rehabilitation procedures]	Across indications	Provision of an overview of initiation, application for or prescription of rehabilitation	Theoretical (perspective: GPs)	• Role of the GP as gatekeeper for access to IMR • Importance of the GP's diagnostic report
Dunkelberg & van den Bussche	2004	[Need for and benefit of medical rehabilitation procedures from a general practitioner’s perspective]	Across indications	Investigation of the attitudes of GPs regarding the need, demand and effectiveness of rehabilitation	Mixed methods: cross-sectional survey (2.110 GPs), interviews (29 GPs) and focus groups (16 GPs)	• GPs have a rather positive attitude towards IMR in general • GPs report communication deficits with cost units
Dunkelberg et al	2002	[The opinion of general practitioners in east and west Germany on rehabilitation]	Across indications	Investigation of the attitudes of GPs on rehabilitation	Quantitative: cross-sectional survey (2.033 GPs)	• GPs have a rather positive attitude towards IMR in general • Approx. 50% of patients who want IMR need it from the GPs' point of view
Fuchs & Chenot	2022	Rehabilitation	Across indications	Provision of an overview of initiation, application for and after care of rehabilitation	Theoretical (perspective: GPs)	• The GP's tasks are to identify needs, support applications and provide follow-up care
Fuchs et al	2017	Fostering needs assessment and access to medical rehabilitation for patients with chronic disease and endangered work ability: protocol of a multilevel evaluation on the effectiveness and efficacy of a CME intervention for general practitioners	Across indications	Evaluation of an assessment-intervention for GPs regarding access to rehabilitation	Study protocol	• A tool could support GPs in identifying rehabilitation needs of their patients
Gohlke et al	2000	[Influence of optimized interface management on the long-term effectiveness of cardiac rehabilitation]	Cardiology	Evaluation of an optimised long-term management on the efficacy of cardiological rehabilitation	Quantitative: prospective controlled trial (1.042 patients)	• Communication problems between GPs and rehabilitation clinics have no influence on the success of rehabilitation after 6 months
Golla et al	2023	[Comprehension and perspectives of the need for medical rehabilitation—results of a german online survey]	Across indications	Examination of the perspectives regarding the need for rehabilitation in the general population	Quantitative: cross-sectional survey (2.041 persons in the general population)	• Almost 80% of the general population see their GP as the first point of contact for rehabilitation-related questions or problems
Hempler	2021	[How do people who have had a stroke, their relatives and experts experience stroke aftercare following completing a medical rehabilitation program? results of a qualitative study]	Neurology	Exploration of the experiences of stroke patients and professionals regarding follow-up care	Qualitative: interviews (2 GPs, 2 neurologists, 2 physiotherapists, 7 patients, 6 relatives)	• From the point of view of GPs, the quality of the discharge report can influence further follow-up care • There are hardly any established communication structures between the professional stakeholders • GPs are described as gatekeeper regarding follow-up care
Hoffmann et al	2018	[Access to inpatient rehabilitation of methamphetamine addiction – barriers and potential for Improvement from the experts' perspective]	Addictive disorders	Exploration of barriers impeding the access to rehabilitation of methamphetamine addicts	Qualitative: interviews and focus groups (8 GPs, 11 addiction counselors, 20 specialists for psychotherapy and psychiatry)	• GPs are seen as gatekeepers across all professions
Hülsemann	1998	[Cooperation between primary care physicians, specialized rheumatologists, hospitals and rehabilitation clinics]	Orthopedics	Examination of the cooperation between primary care physicians, specialized rheumatologists, hospitals, and rehabilitation clinics	Theoretical (perspective: rheumatologists)	• In follow-up care, GPs have the function of monitoring further therapies
Jankowiak	2016	[Intensified involvement of general practitioners in the rehabilitation process—effects on aftercare and professional participation following rehabilitation]	Across indications	Evaluation of an intervention to involve GPs more intensively in follow-up care	Quantitative: cohort study (2.807 patients)	• A pilot project shows positive effects with regard to the identification of a possible need for rehabilitation by GPs
Jankowiak et al	2019	[GPs play a key role in positive effects of rehabilitation]	Across indications	Evaluation of an intervention to involve GPs more intensively in the rehabilitation process	Quantitative: cohort study (2.807 patients)	• GPs' assessments of rehabilitation outcomes are good predictors of return to work
Konrad	2010	[Implementation of aftercare recommendations following inpatient medical rehabilitation]	Orthopedics	Examination of the actual implementation of follow-up recommendations	Qualitative: document analysis and interviews (50 patients)	• GPs play an important role in follow-up care from the patient's point of view • Patients tend to rate the cooperation between GPs and rehabilitation clinics poorly
Krause	2002	[The rehabilitation status of patients with pension insurance from the perspective of GPs compared with the IRES questionnaire]	Across indications	Identification of the rehabilitation needs of GP patients	Quantitative: cross-sectional survey (100 patients) and needs assessment (by 3 GPs)	• In a third of the cases observed, the assessments of GPs and patients regarding the need for rehabilitation do not match
Krischke et al	1997	[Influence of general practitioners on access to medical rehabilitation]	Across indications	Determination of the influence of GPs on behavior on the decisions regarding rehabilitation applications	quantitative: cross-sectional survey (298 GPs) and document analysis (206 medical reports)	• 20% of GPs consider IMR to be unsuccessful in principle • Between 30%and 40% of GPs support IMR even if they very often or always doubt the success • In almost 50% of diagnostic reports, relevant medical findings are missing
Küpper-Nybelen et al	2003	[Changes of risk factors in patients with coronary heart disease after inpatient rehabilitation]	Cardiology	Examination of follow-up care by GPs to reduce risk factors in patients with coronary heart disease	Quantitative: longitudinal survey (1.206 patients)	• Optimizing the interface between GPs and rehabilitation clinics did not improve the long-term effects of IMR
Kusak et al	2006	[Judgements of physicians in practice with different specialities regarding medical rehabilitation in patients with rheumatoid arthritis]	Orthopedics	Examination of the judgements of physicians in practice regarding rehabilitation in patients with rheumatoid arthritis	Quantitative: cross-sectional survey (540 physicians incl. 189 GPs)	• Less than 20% of GPs saw a need for rehabilitation in at least half of their patients. In contrast, individual rehabilitation goals and procedures were rated highly, e.g. functional improvement, physiotherapy
Lachmann et al	1999	[Need for rehabilitation procedures from a GP's point of view]	Across indications	Identification of the GPs perspective on need, demand and effectiveness of rehabilitation	Quantitative: cross-sectional survey (956 GPs)	• GPs estimate no need for rehabilitation in 53% of cases in which patients express a wish for rehabilitation • GPs want direct communication with rehabilitation clinics and cost units
Lachmann et al	2000	[The benefits of rehabilitation from a GP's perspective—a comparison between the old and new federal states]	Across indications	Identification of the GPs perspective on need, demand and effectiveness of rehabilitation	Quantitative: cross-sectional survey (956 GPs)	• GPs report a not inconsiderable under- and over-utilization of rehabilitation by their patient • Positive general attitude of GPs towards IM
Linden et al	2016	[Treatment options for patients with chronic mental disorders in primary care]	Psychosomatics	Investigation of the long term medical care for patients with mental disorders in general practice	Quantitative: cross-sectional survey and diagnostic interviews (307 patients)	• Confirmation of the gatekeeper function of GPs
Muschalla & Linden	2019	[Indication for inpatient psychosomatic rehabilitation in primary care patients with chronic mental disorders and participation impairments]	Psychosomatics	Investigation of the indication for rehabilitation in general practice patients	Quantitative: clinical examinations and document analysis (307 patients)	• Accessing IMR as well as long-term follow-up care is a task for GPs • In 27% of cases, no IMR have yet been carried out, but have now been recommended by a consultant
Muschalla et al	2013	[Medical rehabilitation by general practitioners in patients with chronic mental disorders]	Psychosomatics	Exploration of the role of rehabilitation medicine in general practice	Quantitative: cross-sectional survey (40 GPs and 1.451 patients)	• GPs are gatekeepers for IMR in patients with chronic mental health problems
Pohontsch & Deck	2010	[Overcoming "interface problems" in medical rehabilitation]	Across indications	Investigation of interface problems and optimization potentials	Qualitative: focus groups (78 participants incl. GPs, specialists in private practice, cost bearers, physicians in rehabilitation clinics and patients)	• Rehabilitation physicians report problems in working with GPs due to a lack of time resources and a high level of bureaucracy, among other things • GPs are not necessarily seen by the rehabilitation clinicians as the contact person (for the rehabilitant) for follow-up care
Pohontsch & Deck	2011	[Interface problems in rehabilitative care]	Across indications	Investigation of interface problems and optimization potentials	Qualitative: focus groups (78 participants incl. GPs, specialists in private practice, cost bearers, physicians in rehabilitation clinics and patients)	• cost units, rehabilitation clinics and GPs see deficits in cooperation with regard to follow-up care
Pohontsch et al	2013	[Recommendations for overcoming interface problems in medical rehabilitation of federal pension funds and statutory health insurance]	Across indications	Investigation of interface problems and optimization potentials	Qualitative: focus groups (78 participants incl. GPs, specialists in private practice, cost bearers, physicians in rehabilitation clinics and patients)	• Recommendations for reducing interface problems in IMR: including less bureaucracy, information services for GPs and further training opportunities
Schencking	2009	[Rehabilitation and spa medicine in Germany - General practitioners set the course]	Across indications	Explanation of the role of GPs in initiating IMR	Theoretical (perspective: GPs)	• The central task of GPs in the context of IMR is to initiate rehabilitation
Schliehe	2009	[Office practice physicians and rehabilitation]	Across indications	Discussion on the Influence of GP on the application behavior of patients	Theoretical (perspective: rehabilitation sciences)	• GPs can have an influence on their patients' IMR application behavior • Application procedures should be better coordinated to facilitate the involvement of GPs
Schott et al	2002	[Continuity and the quality of medical care for the cardiac patient—access to rehabilitation and the interface with everyday life]	Cardiology	Investigation of the interfaces in cardiac regarding process continuity	Quantitative: cross-sectional survey (1.056 patients)	• The preparation of patients for IMR should be improved, including the involvement of GPs
Schubert et al	2012	[Access to medical rehabilitation from the viewpoint of practitioners—problems and opportunities for improvement]	Across indications	Exploration of Influencing factors and opportunities for improvement in the access processes to rehabilitation	Qualitative: focus groups (22 healthcare professionals incl. 11 GPs and interviews (51 healthcare professionals incl. 32 GPs)	• Long periods of incapacity for work are an important criterion for GPs when identifying rehabilitation needs • From the GPs' perspective, recognizing the need for IMR is often experience-based
Seger et al	2008	[Perspectives in rehabilitation—general position paper on future trends and challenges in rehabilitation by the health advisory board of the German federal association for rehabilitation]	Across indications	Discussion on future trends and challenges in Rehabilitation	Theoretical (perspective: medical experts)	• GPs have a coordinating role both for the access to IMR and for follow-up care
Stratil et al	2017	Cooperation between general practitioners, occupational health physicians, and rehabilitation physicians in Germany: what are problems and barriers to cooperation? a qualitative study	Across indications	Investigation of the cooperation between GPs, occupational health physicians, and rehabilitation physicians	Qualitative: focus groups (22 GPs, 9 occupational physicians, 12 rehabilitation physicians, 15 rehabilitants)	• Cooperation between GPs and occupational physicians in relation to IMR could be improved from the perspective of both groups
Stratil et al	2017	Optimizing cooperation between general practitioners, occupational health and rehabilitation physicians in Germany: a qualitative study	Across indications	Examination of the optimization of the cooperation between GPs, occupational health physicians, and rehabilitation physicians	Qualitative: focus groups (22 GPs, 9 occupational physicians, 12 rehabilitation physicians, 15 rehabilitants)	• Improving cooperation between GPs and occupational physicians and other relevant stakeholders in relation to IMR could be achieved primarily through direct communication and less bureaucracy
Stratil et al	2018	Image and perception of physicians as barriers to inter-disciplinary cooperation?—the example of German occupational health physicians in the rehabilitation process: a qualitative study	Across indications	Exploration of the image and perception of GPs, occupational health physicians, and rehabilitation physicians	Qualitative: focus groups (22 GPs, 9 occupational physicians, 12 rehabilitation physicians, 15 rehabilitants)	• Negative attitudes can hinder improvement of interprofessional cooperation regarding IMR
van den Bussche & Dunkelberg	2003	[Who should decide on applications for medical rehabilitation?]	Across indications	Examination of the responsibility on deciding about applications for rehabilitation	Quantitative: cross-sectional survey (2.110 GPs)	• 40% of GPs rate their knowledge of IMR and the approval process as good or very good • IMR does not always have a good image among GPs
Vogel et al	1997	[Attitudes of office practice physicians towards medical rehabilitation: an empirical Investigation of the problem of rehabilitation access]	Across indications	Exploration of attitudes of GPs towards rehabilitation	Quantitative: cross-sectional survey (382 GPs)	• The assessment of the importance of IMR from the perspective of GPs varies between indications • 42% of all IMR applications are initiated by patients
Wagener	2002	[General practitioner as moderator]	Across indications	Discussion of the role of GPs in rehabilitation	Theoretical (perspective: GP´s)	• According to social legislation, general practitioners must be involved in the initiation of rehabilitation
Walther et al	2015	[Need for information concerning medical rehabilitation of the federal german pension fund—findings of an online survey of general practitioners]	Across indications	Identification of the need for information of GPs concerning rehabilitation	Quantitative: cross-sectional survey (149 GPs)	• The information needs of GPs on 40 rehabilitation-specific topics surveyed amounted to between 27 and 91%
Walther et al	2015	[Well Informed into rehab? what information have patients before and after rehabilitation received, searched for and missed? results of guided interviews and focus group]	Across indications	Identification of the information status and information needs of patients	Qualitative: focus groups (32 patients) and interviews (24 patients)	• Patients mainly seek information from GPs before starting an IMR
Walther et al	2018	[Need for information about medical rehabilitation of persons with german pension insurance:a written survey]	Across indications	Identification of the information status and information needs of patients	Quantitative: cross-sectional survey (196 patients)	• In the event that patients require an IMR, the majority (77%) would first contact their GP
Weier et al	2020	[General practitioner centered rehabilitation aftercare for chronic back pain]	Orthopedics	Evaluation of an intervention regarding follow-up care in general practice	Mixed methods: longitudinal observational survey (85 patients) and interviews (11 patients, 14 GPs)	• Intensified involvement of GPs in follow-up care shows positive effects in several relevant outcomes
Weier et al	2021	[General practitioner centered rehabilitation aftercare for chronic back pain]	Orthopedics	Evaluation of an intervention regarding follow-up care in general practice	Quantitative: longitudinal observational survey (85 patients)	• GP-centered follow-up care can be integrated easy and effective in existing GP-structures
Werner	2021	[Rehabilitation aftercare works better with the general practitioners]	Orthopedics	Evaluation of an intervention regarding follow-up care in general practice	Quantitative: longitudinal observational survey (85 patients)	• GPs can sustainably strengthen the effects of IMR
Wiesemann et al	2001	[General practitioner aftercare for stroke patients]	Neurology	Examination of the quality of care provided by GPs regarding follow-up care	Quantitative: prospective observational survey and document analysis (131 patients)	• Cooperative follow-up care documented at the interface can demonstrably stabilize the state of health after IMR
Wiesemann et al	2004	[Quality of primary medical care after In-patient stroke rehabilitation—(HANS-Study of GP)]	Neurology	Examination of the quality of care provided by GPs regarding the after care of a rehabilitation	Quantitative: prospective observational survey and document analysis (131 patients)	• Cooperative follow-up care documented at the interface can demonstrably stabilize the state of health after IMR
Winge et al	2002	[Interfaces of rehabilitation: 3 models]	Across indications	Comparison of three models for the description of interfaces in rehabilitation	Theoretical (perspective: rehabilitation system)	• GPs play an important role as gatekeepers in the context of IMR, both in terms of application and follow-up care

*IMR* Inpatient medical rehabilitation, *GP* General practitioner, [*Title*] Translation of an original German-language title

### Results of the thematic analysis

**Fig. 2:**
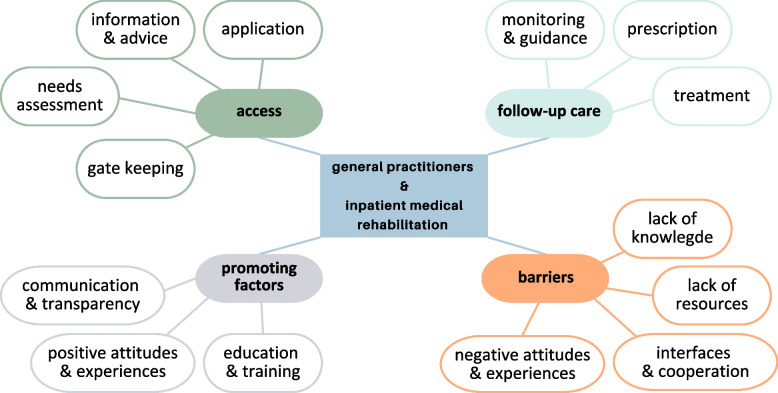
Thematic map

A total of 14 themes were identified that relate to the research questions of this scoping review. These themes were assigned to 4 topic areas, which are presented below. Figure 2 provides an overview of the topic areas and themes.

The themes “needs assessment” and “monitoring & guidance” are addressed most frequently in the publications. The themes “treatment” and “prescription” appear less frequently. Table 3 shows how often the respective themes are addressed in the included publications.

**Table 3:** Themes addressed in the included publications (*n* = 54 publications)

Topic areas	Themes	Frequencies	Percentages
Access	Gate keeping	15	28%
	Needs assessment	23	43%
	Information & advice	10	19%
	Application	17	31%
Follow-up care	Monitoring & guidance	19	35%
	Prescription	5	9%
	Treatment	4	7%
Barriers	Lack of knowledge	10	19%
	Lack of resources	8	15%
	Interfaces & cooperation	17	31%
	Negative attitudes & experiences	14	26%
Facilitators	Education & training	10	19%
	Communication & transparency	9	17%
	Positive attitudes & experiences	11	20%

### Involvement: access to IMR

One form of GPs' involvement in the IMR identified in the literature is access to the IMR. The following four themes were assigned to this topic area.

#### Gate keeping

Various publications attribute the gatekeeping function of GPs in the context of access to IMR [10, 23–33]. This function is not only politically intended [24], but is also partly described by GPs themselves and medical colleagues [25] and is closely linked to some of the defining characteristics of the GP profession itself. These include the fact that GPs treat many patients with chronic illnesses and/or impairments to participation [6, 27]. In addition, they have a special responsibility for patients due to the exceptional nature of their relationship with their patients and the general position of the GP in the healthcare system [31]. Moreover, GPs are also described as the first point of contact for any rehabilitation needs or requests from patients [10, 31]. This is confirmed by patient surveys [1, 34].

#### Needs assessment

An important task for GPs in accessing IMR is to identify eligible patients at the right time for IMR [10, 23–25, 35], in order to initiate IMR as early as possible and thus increase the likelihood of successful treatment [36, 37], but also to avoid overuse [31]. One study reports that the quality of GPs' assessments of rehabilitation needs is good [30]. In order to optimize and facilitate this identification of needs, information mediation has already been carried out [38] as well as tools tested [2, 10, 39, 40]. Some publications report criteria for recognizing needs, but these can vary depending on the diagnosis. Among other things, functional limitations [2], increasing periods of incapacity for work [6, 40], age [27], GPs' subjectively perceived benefits of IMR [41], patients' expectations [39], or the assumption that patients' chronic complaints will be alleviated [42] are mentioned. The frequency of the identified needs for IMR varies in the publications depending on the indication or sample [10, 18, 27, 41–47].

#### Information & advice

Another form of GPs' participation is the information or advice provision to patients before applying to the IMR [18, 45]. These services take place both proactively, i.e. initiated by GPs [23, 25, 48], and reactively, i.e. initiated by patients [34, 41, 47–50].

#### Application

GPs play an important role in the application process for IMR by providing general support in preparing and compiling the application forms [6, 29, 31, 32, 35, 39, 42, 45, 47–49, 51]. Furthermore, GPs prepare a so-called "Befundbericht” (diagnostic report) [6, 24, 40, 42, 45, 48, 49, 51–53], regardless of the cost unit to which this IMR is applied for. In addition to rehabilitation-relevant diagnoses, the content of the report includes the associated functional disorders that justify rehabilitation as defined by the International Classification of Functioning, Disability and Health (ICF) [54]. If the cost units approve IMR, the task of GPs is also to determine the physical and mental ability or patients for IMR [6, 30].

#### Involvement: Follow-up care after IMR

Another topic area in which GPs are involved in the IMR is follow-up care after rehabilitation. The following three topics were identified in this regard.

#### Monitoring & guidance

According to the publications included, one of the main tasks of GPs following IMR is monitoring and guidance. GPs guide the implementation of the recommendations that patients receive at the end of their inpatient stay in the rehabilitation clinics [27, 36, 41, 53, 55–60]. Accordingly, GPs' tasks are explicitly seen as controlling [7, 57, 61, 62] and coordinating the (outpatient) follow-up care services [30, 31, 48, 51, 56, 63, 64].

#### Prescription

The scope of coordination also includes the recommendation-based or GP-assessed prescription of medications and aids [64], referral to outpatient medical specialists [64] and therapists [6, 63], as well as the prescription of training [6, 7, 24], which all should ensure or reinforce the rehabilitation effects.

#### Treatment

In some of the included publications, specific treatments by GPs are mentioned as follow-up care. These relate to diagnosis [56, 57], medication [63], or motivating and ensuring patient compliance to pursue the rehabilitation goals in the long term [41, 63].

### Barriers

A further research interest targets barriers to the involvement of GPs in IMR reported in the literature. Four themes were assigned to this topic area as part of the analysis.

#### Lack of knowledge

Some publications report knowledge deficits among GPs. These relate to the requirements and processes of an IMR application, e.g. with regard to the legal requirements for IMR [23, 40, 65], the approval criteria and revocation options [18, 49, 66], recognizing the need for IMR [66, 67], and the rehabilitation prognosis [66]. Moreover, general knowledge deficits are reported, e.g. regarding the purposes and objectives of IMR [2, 23, 40, 50], the range of procedures of IMR [2, 40, 47, 49, 65], single procedures [23], or rehabilitation clinics [23]. One publication also reports knowledge deficits regarding follow-up care [65].

#### Lack of resources

Another area of barriers relates to scarce resources of GPs. On the one hand, budget restrictions can make it difficult to prescribe aids and remedies like physiotherapy for needs-based follow-up care. Due to the limited number of possible prescriptions of GPs in Germany, this can lead to difficulties in follow-up care by GPs if, for example, budgets have already been exhausted [64]. On the other hand, time expenditure or lack of time is described as a barrier for needs assessments and IMR applications [2, 25, 40, 49], follow-up care [56, 64], and for the communication with cost units and rehabilitation clinics [51, 53].

#### Interfaces & cooperation

Difficulties regarding the interfaces of IMR and the cooperation between the stakeholders involved are described from various perspectives (GPs, cost units, rehabilitation clinics, outpatient specialists, patients). The difficulties encompass lacking or poor communication between outpatient care providers [40, 51, 64], cost units [2, 23, 40, 41, 49, 67], rehabilitation clinics [2, 18, 51, 53, 56, 62, 64, 67], and GPs. A lack of transparency between the stakeholders is also reported [18, 31, 40, 67]. Some publications identify uncertainties and inconsistencies regarding responsibilities [25, 31, 40, 45, 53], approaches and understandings, e.g. concerning specific terms [68], medical approaches [31], and classification systems like ICF and the International Classification of Diseases (ICD) [6, 40].

#### Negative attitudes & experiences

Negative attitudes and experiences of GPs can be problematic for GPs' involvement in IMR. In some cases, a perceived lack of influence on approval decisions is reported by GPs [42, 49, 67]. Skepticism towards IMR in general [2, 18, 39, 40, 42, 49, 63] and negative attitudes towards stakeholders involved (e.g. cost units, rehabilitation physicians) are identified as barriers [2, 49, 52, 53, 67, 69]. For example, some GPs consider the high staff turnover in the rehabilitation clinics to be problematic [67]. Another frequently discussed problem area is the experience of time-consuming and sometimes unreasonable bureaucratic procedures and tasks, such as applying for an IMR [2, 25, 39, 40, 49, 51, 53, 67, 70].

### Facilitators

In addition to barriers, facilitators promoting the involvement of GPs in IMR were identified in the publications. These were assigned to the following three themes.

#### Education & training

Education and training for GPs that serve to impart knowledge and skills are highlighted as beneficial. These benefits affect both basic medical training [2, 31, 68] and further training regarding benefits and aims of IMR [18, 23, 31, 40, 70]. The positive effect of information material on requirements and structures of IMR for GPs is also discussed in this context [40, 49, 65, 70, 71].

#### Communication & transparency

The possibility of direct communication between GPs and other stakeholders (cost units, rehabilitation clinics, outpatient specialists) was identified as a facilitator. This explicitly applies to the possibility of verbal contact by telephone calls [18, 45, 49, 65], communication via e-mail [65, 68], or the general possibility of communication that goes beyond the official reports [32]. A more transparent presentation of the decision-making process on the application for IMR, including greater involvement of GPs, can have a positive impact on the general involvement of GPs in IMR [18, 40, 45, 49, 65]. From the GPs' point of view, it would be positive to have options for further explanations and comments in the application forms and the GPs' diagnostic report [49, 65, 68]. Regarding the discharge report from the rehabilitation clinic, a direct and detailed approach to the GPs by the rehabilitation physicians is perceived as helpful [6, 18, 64].

#### Positive attitudes & experiences

In contrast to negative attitudes and experiences, which have already been identified as barriers, are positive attitudes and experiences of GPs in the context of IMR described as facilitators. This includes attitudes and experiences in relation to the approval process [23], treatment in the rehabilitation clinics [45, 63], outcomes of the IMR [18, 23, 37, 41, 42, 47, 63], follow-up care [18, 68], or IMR in general [18, 23, 41, 43, 46, 47].

## Discussion

The aim of the study was to provide an overview of GPs' involvement in IMR in Germany as well as to identify barriers and facilitators promoting this involvement. GPs are primarily involved in two areas: access to IMR and follow-up care. Barriers and facilitators relate to both personal and structural aspects. There were no aspects that emerged and disappeared over a period of several years or that have changed fundamentally over the years. For example, a study from 1997 shows that some GPs are rather skeptical about IMR and some rather positive and that diagnostic reports are often incomplete [42]. Both aspects, the existence of different attitudes towards IMR [63] and the problem of incomplete diagnostic reports [52], were also identified in studies eleven and 21 years later.

### Access to IMR and follow-up care

GPs' involvement in providing access to IMR is frequently described and justified in the identified publications. In Germany, patients [1], the German pension insurance [13], political decision-makers [12], and, in some cases, GPs themselves [48] highlight and encourage this involvement. There is empirical evidence that GPs' involvement is also beneficial for patient outcomes. For example, it has been reported that GPs' assessments of rehabilitation outcomes are good predictors of reintegration into working life [37]. In general, GPs have access to a broad spectrum of patients, including vulnerable groups (e.g., lower socio-economic background) [72]. Thus, GPs can contribute to the goal of needs-based and early access to rehabilitation of all patient groups and prevent both overuse and underuse, which are considered reasons for unstable long-term effects of IMR [40, 73]. The gatekeeper function of GPs [74] is not particularly surprising, as GPs perform this function in many international healthcare systems. In Sweden, for example, GPs also play an important role in patients’ access to rehabilitation. As Sweden has a highly centralized healthcare system, GPs are often responsible for organizing and referring patients to rehabilitation services, whether in hospitals or in outpatient rehabilitation centers [75]. In Austria, GPs have a gatekeeper function with regard to access and follow-up care [76]. Studies from the United Kingdom [77], Denmark [9], Norway [78], and Canada [79]. report different effects of attempts to optimize GPs' involvement in this context. Nevertheless, differences in the respective healthcare systems could have an influence on the specific role of GPs in accessing IMR. These concern, for example, the transfer of patients to the IMR, which in Germany often starts from the home environment [3]. In other countries, such as the United Kingdom, patients are rather transferred from hospitals [80]. In these different situations, the involvement of GPs regarding access to IMR can differ, e.g. when applying for rehabilitation. In this context, the active role of patients in accessing IMR in Germany [31] is important. In France, for example, applications for rehabilitation are made almost solely by clinicians [76]. In the Netherlands, they are primarily submitted by occupational physicians [77].

Follow-up care is recognized as highly important to ensure prolonged effects of rehabilitation [81]. Therefore, once patients leave the IMR, there is a whole range of follow-up care services. On the one hand, GPs' involvement in follow-up care is due to their gatekeeper function since GPs provide, for example, prescriptions for physiotherapy or medication [63]. On the other hand, the general characteristics of the GP role and the patient-doctor relationship (long-term, knowledge of the life situation and health biography, familiarity, and trust) are very well suited for monitoring long-term effects and helping to achieve rehabilitation goals [71]. This is particularly important considering the difficulty of maintaining IMR effects over a long period of time [82].

### Facilitators and barriers

Some aspects of GPs' involvement in IMR can operate as both facilitators and barriers, depending on the context. Examples are the attitudes and experiences of GPs regarding IMR. Surveys revealed that the more negative the general attitude of GPs towards IMR, the more reluctant they are to provide rehabilitation recommendations to patients, whereas a positive general attitude towards IMR correlates significantly with a positive assessment of individual aspects of rehabilitation [18]. Another example is the interface and communication aspects between GPs and other stakeholders, such as rehabilitation clinics or insurances. This aspect is particularly important for IMR because a large number of stakeholders can be involved in the rehabilitation process [7]. Despite frequently reported potential for optimization [67], direct communication channels and transparency are highlighted as beneficial by various stakeholders [64, 65, 68].

Some publications report a lack of GPs' knowledge regarding IMR [65, 67]. Evidence of such knowledge deficits can also be found in the international literature, for example, on the basis of self-attributions by Australian [83] or Iranian [84] GPs. Despite efforts to increase information and knowledge among GPs and to sensitize GPs about rehabilitation-related topics [11, 37], there is still a need for improvement. In Germany, rehabilitation-related topics are often given little attention in medical studies [66]. Also, there are only a few opportunities for further and advanced training, especially for GPs [75]. More training opportunities are crucial, considering the fact that GPs have many potential rehabilitants among their patients and the identification and coverage of needs is a central task. In addition, the aspect of training and education has been identified as a facilitator in publications of the review [31, 40, 70],

A lack of resources is described as a problem in the GP context and in the German healthcare system as a whole [85]. Internationally, a lack of resources is reported as a barrier, for example, in the United Kingdom in relation to time constraints in vocational rehabilitation [86]. Resources are an important aspect, but also involvement in IMR is a defined area of responsibility for GPs in Germany. Any unnecessarily time-consuming processes and tasks need to be scrutinized. One example is the bureaucratic effort involved in applying for an IMR, which is sometimes criticized by GPs [40].

### Recommendations for practice and need for further research

Research results suggest a more active involvement of GPs in the described areas of IMR [19]. In order to ensure and, if necessary, optimize this involvement, the barriers identified in this scoping review need to be addressed. There have already been attempts to address the barriers, e.g., through practice-oriented research projects in the areas of information [11], awareness-raising [19], or cooperation [70]. However, various problems still appear to be relevant, requiring further efforts. The training and education of physicians could provide further offers for interested parties, e.g., concerning the recognition of a need for rehabilitation, or the structures of IMR, and the range of services offered by rehabilitation clinics. Lack of resources, as identified as a barrier in the review, is a general problem in most healthcare systems [87]. Good solutions need to be found to make the best possible use of GPs' role in IMR, but not to overburden them. One solution could be to minimize the bureaucratic burden in the context of IMR, which is criticized by GPs [16].

The included publications show a broad spectrum of methodological approaches and perspectives. The range of publication years shows that the topic has been researched from the late 1980 s to the present day. Nevertheless, some empirical evidence is now over 20 years old. Changes in the healthcare system, such as the role of patients and thus a changing doctor-patient relationship [87], or modifications in the rehabilitation system [88] could justify a renewed and up-to-date examination of the identified topics. In addition, indication-specific studies are often lacking. It can be assumed that issues such as the identification of needs or adequate follow-up care differ between different IMR-indications. For example, special features are conceivable in the psychosomatic field, which are different in the orthopedic field. The psychosomatic area in particular should be the focus of more in-depth scientific research, as it can be assumed that related needs are increasing in the future [4]. Evidence shows that GPs are particularly uncertain about diagnostics, needs recognition, and IMR options for psychosomatic issues [52]. Research on IMR could also benefit from comparisons between international systems to identify possible opportunities for adoption.

### Strengths and limitations

This review summarizes for the first time the state of research on the involvement of GP in IMR in Germany. By proceeding according to an established methodological approach, the results are based on a robust methodological basis. A wide range of literature was included and analyzed, which suggests that relevant literature for answering the research questions was identified. Furthermore, a broad understanding was used to not limit the variety of aspects identified in the literature. In addition, deciding whether a publication or text fragment matches the inclusion criteria is a subjective process. However, these decisions were made intersubjectively and transparently by bringing together researchers from different disciplines and discussing relevant decisions within the research team.

One major limitation is the national limitation of the review's results. In conjunction with the peculiar characteristics of the German rehabilitation system, a general transferability of the results to international systems is challenging without further ado. Although this scoping review employed various databases, only literature that was accessible to the research team was used. Four publications that would have been included after title and abstract screening could not be fully screened in full-text due to unavailability. This could have an influence on the results. The authors, however, estimate this influence to be small.

## Conclusion

Rehabilitation is linked to other outpatient and inpatient medical areas in various ways. Close cooperation and networking with outpatient primary care is crucial for rehabilitation in Germany and in some other countries. Thus, the rather lengthy rehabilitation processes can be initiated, executed, and followed up as smoothly as possible. The results of this scoping review confirm the important position of GPs in access and follow-up care for IMR in Germany and at the same time reveal a variety of barriers to GPs' involvement. In order to maintain and optimize GPs' position, further scientific research and efforts based on this in practice are necessary. All relevant stakeholders should be involved here.

## Data Availability

No datasets were generated or analysed during the current study.

## Abbreviations

GP: General practitioner
ICD: International Classification of Diseases
ICF: International Classification of Functioning, Disability and Health
IMR: Inpatient medical rehabilitation
JBI: Joanna Briggs Institute
